# Supplementary material for: Evaluation of the adverse events following immunization surveillance system, Ghana, 2019
Source: PLoS One. 2022 Mar 1;17(3):e0264697. doi: 10.1371/journal.pone.0264697 (PMC8887767; doi:10.1371/journal.pone.0264697)
Supplement: Attachment — Submitted filename: Response to Reviewers.pdf [file pone.0264697.s005.pdf]

The Academic Editor
PLOS ONE

Dear Sir

Evaluation of the Adverse Events Following Immunization Surveillance System, Ghana, 2019 (PONE-D-21-21870)
Thank you for the review and providing comments to improve our manuscript. The table below are responses to specific comments made by the reviewers.

COMMENT	CORRECTIONS MADE
**RESULTS**	
The case definitions, should be within the Methods Section.	The AEFI case definitions, have been put under data collection within the Methods Section. This can be seen in lines 132 to 142.

Thank you for your comments and inputs.

Sincerely,

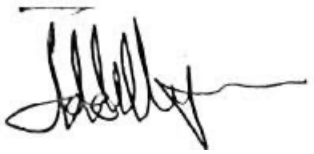

………………………………..
