# Supplementary material for: Evaluation of the adverse events following immunization surveillance system, Ghana, 2019
Source: PLoS One. 2022 Mar 1;17(3):e0264697. doi: 10.1371/journal.pone.0264697 (PMC8887767; doi:10.1371/journal.pone.0264697)
Supplement: Attachment — Submitted filename: Reviewarticle.docx [file pone.0264697.s002.docx]

Abstract:

Line 40: The three updates within last decade appears not to be included in the main body of text

Line 43: ‘All (16/16) Community Health Nurses interviewed indicated “fear of being victimized”’ doesn’t connect to the second part of the sentence; needs to be made more clearer

Introduction:

Line 54: “Vaccine safe and effective; however adverse events following immunization”: this is contradictory; although I agree vaccines are generally safe; perhaps add the word, Generally vaccines are safe and effective;

Also it would be beneficial to add something that vaccines undergo extensive safety testing during clinical trials but rare events can be detected through post marketing surveillance

Line 60 ; serious and non-serious should not be confused with severity; based on the outcome of the event (e.g. hospitalisation, death); seriousness is not based on severity; this sentence should be corrected.

Line 66: vaccine hesitancy is a public health issue; I would suggest replacing the term “concerns” with vaccine hesitancy:

67: There should be an explanation on why surveillance on immunization is essential; I.e.: to build trust; to reassure the public that AEFIs are being monitored and actions to reduce risks are being taken

Line 69:AEFI to AEFIs

Line 69: Under reporting is a well-known with routine reporting systems

Line70: the term routine is preferable to passive

Line 70: HICs also rely on routine systems; under reporting is a general limitation of passive/routine systems; but is there any other reason that LMICs have additional underreporting?

Line 70 -72: perhaps rephrase to emphasize the link: underreporting was addressed via the GVSI. One of the targets of… was set.

Materials and methods

Line 93: is there a reference for CDC guidelines for evaluating ?

Line 94: how were these guidelines adapted? What were the quantitative and qualitative data that was collected?

Line 98: Study area paragraph: The evaluation was made through interviewing stakeholders in this region? And reviewing reports in this district? Perhaps clarify what resources in this area was used to do the evaluation.

Line 125: I don’t think the term detect can be applied here; perhaps just state that caregivers/ vaccinees report to health facilities. How do they report? Using a reporting form?

Line 126: What is meant by manage reports? They receive and code them? And they transfer the reports?

Line 153: title says data collection and analysis, but no analysis was described: perhaps expand on the analysis or remove analysis from title.

Also please make data sources clearer :by stating five different sources of data was collected data included: a) interviews .. b) case-based forms; c) IDSR reports; d) DHIMS2; e) TAC.VBP reports

Line 159: explanation of what TAC-VBP reports are?

Line 165: explain what is meant by hypothesis generation; is this signal detection?;

Line 199; prefer to use the word understood rather than knew

Line 205 section on performance of the surveillance system in meeting objectived: objective were; 1) detect, correct and prevent immunization error-related AEFIs; 2)

identify problems with vaccine lots or brands leading to vaccine reactions, 3) prevent false

blame arising from coincidental adverse events, 4) maintain confidence by properly responding

to community concerns, 5) generate new hypotheses about vaccine reactions and estimate rates

of occurrence of AEFIs.

Use subtitles to indicate the results of assessment of each of these objectives;

Line 208: would it be better to say 463/476 (97%) of all serious cases were investigated?

Line 209: suggest to rephrase replace the word exonerate; By investigating deaths that followed vaccination and performing causality assessments the system has been able to prevent misinformation speculating that the vaccine attributed to the deaths? Also link tis to the objective of prevent false blame arising from coincidental adverse events

Line 211: link lot/batch number to objective: identify problems with vaccine lots or brands leading to vaccine reactions

Line 212: I would replace the word victim; perhaps individual;

Line 212: who paid for the management of the AEFIs?

Line 214: it is difficult to get an incidence rate with spontaneous reporting as there is always under reporting. Even if exposure information is known, the actual number of AEFIs collected through spontaneous reporting is an under estimation of incidence. Active surveillance methods are needed to estimate the incidence

Line 212-213: does this refer to signal ( hypothesis generated)? This needs to be made more clearer

Line 215: how was the usefulness assessed? Was this through interviews? Where did this information come from?

Line 223-224: case definition of AEDI reports?,

Line 224: please do not use the word victim; an individual is suspected to have AEFI; causality is not determined; the word victim gives a negative connotation, when most of the time benefits of the vaccine outweigh the risk

Line 225-230: was 15 minutes to fill a report and investigations into serious cases considered to be simple?

Line 280-281: was this a human error or a problem with the tools?

Line 308: in Ghana is this the case, do caregivers have to go back to the facility to report? Or are there tools in place to allow patient/caregiver reporting?

Line 344: full stop (.) after system

Line 349: does Whatsapp platforms compromise the completeness of reports?

Another limitation is recall bias in interviews; as study was conducted in 2019 , investigating from year 2014.

Generally: Objectives and measurables need to be made clear; difficult to connect methodology to results: layout needs revision
