# Supplementary material for: Evaluation of the adverse events following immunization surveillance system, Ghana, 2019
Source: PLoS One. 2022 Mar 1;17(3):e0264697. doi: 10.1371/journal.pone.0264697 (PMC8887767; doi:10.1371/journal.pone.0264697)
Supplement: Attachment — Submitted filename: PONE-D-21-21870.docx [file pone.0264697.s003.docx]

PONE-D-21-21870

Title: Evaluation of the Adverse Events Following Immunization Surveillance System, Ghana, 2019

The authors present an evaluation of Ghana’s AEFI surveillance system (i.e. does the system meet the programme’s objectives) using an adaptation of a CDC tool. Vaccine safety is an important area of research and underserved in sub-Saharan Africa and the study has the potential to add to the knowledge base. Unfortunately, in its current form, the manuscript does not meet the criteria for publication.

The overall methodology is very unclear, although some study procedures are described. At present it is difficult to relate the methods to the objectives and results.

This could be addressed by the following:

Define the database.

Proper introduction to and explanation (and referencing) of the tool and how this was applied (e.g. stakeholder interviews and record reviews with reference to the table). I sense that there are both quantitative and qualitative elements in the tool.

How this was applied to evaluate the objectives of the AEFI programme, which is the aim of study.

Certain attributes are assessed (presumably part of the evaluation tool) but these are not explicitly related to the objectives of the AEFI surveillance system.

It may be useful to define the outcome terms in the text when you report the results e.g. PPV of the system detecting the number of vaccine-related AEFI etc.

Reviewer 2 has offered a very thorough summary with constructive suggestions.

Limitation: Immunization rates are low (page 5) – how could this affect interpretation of the results?

Line 333: are these results presented? Did you limit the study to AEFI related to EPI only?
