# Supplementary material for: Evaluation of the adverse events following immunization surveillance system, Ghana, 2019
Source: PLoS One. 2022 Mar 1;17(3):e0264697. doi: 10.1371/journal.pone.0264697 (PMC8887767; doi:10.1371/journal.pone.0264697)
Supplement: Attachment — Submitted filename: Response to Reviewers.docx [file pone.0264697.s004.docx]

The Academic Editor
PLOS ONE

Dear Sir

**Evaluation of the Adverse Events Following Immunization Surveillance System, Ghana, 2019 (PONE-D-21-21870)**

Thank you for the review and providing comments to improve our manuscript. The table below are answers or responses to specific comments made by the reviewers.

| **COMMENT** | **COORECTIONS MADE** |
| --- | --- |
| **ABSTRACT** | |
| Line 40: The three updates within last decade appears not to be included in the main body of text | The monthly vaccination report and case-based form were modified to accommodate new indicators. The AEFI case definition was also altered from a definition that implicated the vaccine use to a definition that the AEFI may or may not be caused by the vaccine. This has been included in lines 493 to 496 at the results section. |
| ‘All (16/16) Community Health Nurses interviewed indicated “fear of being victimized”’ doesn’t connect to the second part of the sentence; needs to be made more clearer | The sentence has been revised to build the connection between the two phrases to make the meaning of sentence clearer. This can be seen in line 45 to 48. |
| **INTRODUCTION** | |
| “Vaccine safe and effective; however adverse events following immunization”: this is contradictory; although I agree vaccines are generally safe; perhaps add the word, Generally vaccines are safe and effective; | The word “Generally” was introduced to the sentence in line 60. |
| Also, it would be beneficial to add something that vaccines undergo extensive safety testing during clinical trials but rare events can be detected through post marketing surveillance | The sentence was improved by adding that vaccines undergo extensive safety monitoring prior to its usage in immunization programs. However, no vaccine is entirely without risk. This can be found in lines 60 to 63. |
| Serious and non-serious should not be confused with severity; based on the outcome of the event (e.g. hospitalisation, death); seriousness is not based on severity; this sentence should be corrected. | This classification is based on WHO guidelines. The sentence was altered to “Based on the outcome of the event, the World Health Organization (WHO) has classified AEFIs as Serious and Non-serious which is used for regulatory classification”. This can be seen in lines 68 to 70. |
| Vaccine hesitancy is a public health issue; I would suggest replacing the term “concerns” with vaccine hesitancy: | The term “concerns” was replaced with “vaccine hesitancy” shown in line 75. |
| There should be an explanation on why surveillance on immunization is essential; I.e.: to build trust; to reassure the public that AEFIs are being monitored and actions to reduce risks are being taken | An explanation to why surveillance on immunization safety is crucial has been added to the sentence. This can be seen in line 76 to line 85 |
| AEFI to AEFIs | AEFI was changed to AEFIs in line 86. |
| The term routine is preferable to passive | The term passive surveillance was changed to routine surveillance in line 87. |
| HICs also rely on routine systems; under reporting is a general limitation of passive/routine systems; but is there any other reason that LMICs have additional underreporting? | Poor pharmacovigilance infrastructure was stated as another reason that LMICs have additional underreporting in line 88. |
| Perhaps rephrase to emphasize the link: underreporting was addressed via the GVSI. One of the targets of… was set. | The sentence was rephrased as “To address the issue of underreporting the WHO therefore instituted the Global Vaccine Safety Initiative, which set out indicators primarily for monitoring case reporting” for emphasis as seen in lines 88 to 90. |
| **METHODS** | |
| Is there a reference for CDC guidelines for evaluating? | The reference for the CDC guideline has been added to line 136. |
| How were these guidelines adapted? What were the quantitative and qualitative data that was collected? | How the CDC guidelines was adapted has been explained in lines 136 to 138. |
| Study area paragraph: The evaluation was made through interviewing stakeholders in this region? And reviewing reports in this district? Perhaps clarify what resources in this area was used to do the evaluation. | The resources in the area used to do the evaluation has been explained in lines 122 to 124. |
| I don’t think the term detect can be applied here; perhaps just state that caregivers/ vaccinees report to health facilities. How do they report? Using a reporting form? | The phrase caregivers/vaccinees detect was changed caregivers/vaccinees report adverse events to health facilities. The health facilities record reported events using a standard case reporting form and submits the report to the District Health Directorate. This has been stated in lines 306 to 308 |
| Line 126: What is meant by manage reports? They receive and code them? And they transfer the reports? | How reports are managed has been explained in lines 308 to 312. |
| Line 153: title says data collection and analysis, but no analysis was described: perhaps expand on the analysis or remove analysis from title. | The title “data collection and analysis,” has been changed to “data collection tools and methods”. Nonetheless, Table 1 shows how data collected on attributes was analysed. |
| Also please make data sources clearer: by stating five different sources of data was collected data included: a) interviews .. b) case-based forms; c) IDSR reports; d) DHIMS2; e) TAC.VBP reports | From lines 232 to 235 the sources of data were listed as suggested. |
| Explanation of what TAC-VBP report is?  Line 165: explain what is meant by hypothesis generation; is this signal detection?; | TAC-VBP reports explained as Technical Advisory Committee for Vaccines and Biological Products. |
| **RESULTS** | |
| Prefer to use the word understood rather than knew | The word knew has been changed to understood in line 282. |
| Section on performance of the surveillance system in meeting objectives: objective were; 1) detect, correct and prevent immunization error-related AEFIs; 2)  identify problems with vaccine lots or brands leading to vaccine reactions, 3) prevent false  blame arising from coincidental adverse events, 4) maintain confidence by properly responding  to community concerns, 5) generate new hypotheses about vaccine reactions and estimate rates  of occurrence of AEFIs.  Use subtitles to indicate the results of assessment of each of these objectives; | For the section on performance of the surveillance system in meeting objectives, all findings have been grouped under the subtitles which are the 5 objectives of the system. This can be seen in lines 336 to 362. |
| Would it be better to say 463/476 (97%) of all serious cases were investigated? | This has been corrected in line 347. |
| Suggest to rephrase replace the word exonerate; By investigating deaths that followed vaccination and performing causality assessments the system has been able to prevent misinformation speculating that the vaccine attributed to the deaths? Also link this to the objective of prevent false blame arising from coincidental adverse events | the phrase the system has been able to exonerate EPI has been revised to the system has been able to prevent misinformation speculating that the vaccine attributed to the death. Seen in line 351. |
| I would replace the word victim; perhaps individual; | The word victim was replaced with individual in line 357 |
| Who paid for the management of the AEFIs? | Management of the AEFIs is paid for by the EPI, stated in line 357 |
| Does this refer to signal (hypothesis generated)? This needs to be made more clearer | Hypothesis generation here means to assign possible causes to the reaction. |
| how was the usefulness assessed? Was this through interviews? Where did this information come from? | How usefulness was assessed and the sources of information for this assessment has been explained from lines 364 to 368. |
| Please do not use the word victim; an individual is suspected to have AEFI; causality is not determined; the word victim gives a negative connotation, when most of the time benefits of the vaccine outweigh the risk | The word victim was changed to individual in line |
| In Ghana is this the case, do caregivers have to go back to the facility to report? Or are there tools in place to allow patient/caregiver reporting? | As at the time this evaluation was done in January 2019, caregivers have to go back to the facility to report AEFI. Later in 2019, a MedSafety mobile application was developed. This is still being piloted in-country and its implementation is not countrywide. |
| Does Whatsapp platforms compromise the completeness of reports? | To prevent issues of incomplete reports, all reports passed through WhatsApp platforms must be accompanied by a case-based form. Nonetheless this gives room for human negligence which can still lead to incomplete report. This was explained in lines 556 and 557 |
| Another limitation is recall bias in interviews; as study was conducted in 2019, investigating from year 2014. | Study limitation was added in lines 576 to 581. |
| Proper introduction to and explanation (and referencing) of the tool and how this was applied (e.g. stakeholder interviews and record reviews with reference to the table). I sense that there are both quantitative and qualitative elements in the tool. | An introduction to and explanation (and referencing) of the tool and how this was applied has been included in lines 135 to 138. |
| Certain attributes are assessed (presumably part of the evaluation tool) but these are not explicitly related to the objectives of the AEFI surveillance system. | Though some attributes are not directly related to the study objectives, they were assessed to understand operations of the system and to explain or discuss findings seen in the attributes of the surveillance system. For instance, knowledge was assessed to understand its relation to underreporting or incomplete reporting, |
| It may be useful to define the outcome terms in the text when you report the results e.g. PPV of the system detecting the number of vaccine-related AEFI etc. | Explanations to the outcome terms are in the CDC guidelines referenced in this manuscript. However, PVP has been explained in line 461 as its application is different from that of infectious disease surveillance systems. |
| Are these results presented? Did you limit the study to AEFI related to EPI only? | AEFI surveillance in Ghana is limited to EPI. However there were recommendations to the EPI after this evaluation for integration of other units that use vaccinations like the Port Health (for Yellow fever vaccination) and Maternal health Unit (for tetanus vaccination) |

**General Comments:**

*Generally, Objectives and measurables need to be made clear; difficult to connect methodology to results: layout needs revision.*

**Correction**

The objective of the study was to assess the attributes of Ghana’s AEFI surveillance system, its usefulness and to determine whether the system is meeting its objectives. This was stated in the last sentence of the introduction. However, to connect these objectives to the methods and results, sub-headings (for each objective) have been introduced in the method and results section to improve consistency.

Also, the layout of the report has been revised for clarity. The operations of the system has been moved to the results section. For the assessment of system meeting its objectives, all findings have been grouped under the subtitles which are the 5 objectives of the system.

Additional requirements:

*1.) Please ensure that your manuscript meets PLOS ONE's style requirements, including those for file naming. The PLOS ONE style templates can be found at* <https://journals.plos.org/plosone/s/file?id=wjVg/PLOSOne_formatting_sample_main_body.pdf>

and

<https://journals.plos.org/plosone/s/file?id=ba62/PLOSOne_formatting_sample_title_authors_affiliations.pdf>

**Response**

The format of the manuscript is revised according to the PLOSE ONE style

Thank you for your comments and inputs

Sincerely,

Joseph Asamoah Frimpong
